# The Role of Endometrial Microbiota in the Pathogenesis of Chronic Endometritis: A Systematic Review and Meta-Analysis

**DOI:** 10.3390/biomedicines14040871

**Published:** 2026-04-10

**Authors:** Angela Vidal, Anaïs Y. Kilian, Vithusha Vinayahalingam, Branislav Zagrapan, Janna Pape, Tanya Karrer, Michael von Wolff

**Affiliations:** 1 Division of Gynaecological Endocrinology and Reproductive Medicine, Women’s University Hospital, Inselspital Bern, University of Bern, 3012 Bern, Switzerland; anaiskilian.md@gmail.com (A.Y.K.); janna.pape@insel.ch (J.P.);; 2 Division of Gynaecological, Kantonsspital Baden, 5404 Baden, Switzerland; vithusha19@hotmail.com; 3 Institute of Tissue Medicine and Pathology, University of Bern, 3008 Bern, Switzerland; branislav.zagrapan@unibe.ch; 4 Medical Library, University Library, University of Bern, 3012 Bern, Switzerland

**Keywords:** chronic endometritis, endometrial microbiome, dysbiosis, infertility, RIF, RPL, *Lactobacillus*

## Abstract

**Background:** Chronic endometritis (CE) is a subtle, often asymptomatic endometrial inflammation marked by CD138^+^ plasma cell infiltration and linked to recurrent implantation failure (RIF), recurrent pregnancy loss (RPL), and unexplained infertility. Emerging evidence implicates endometrial microbiome dysbiosis in CE. **Objective:** To systematically review and conduct meta-analysis on the association between CE and endometrial microbiome alterations and their reproductive implications. **Methods:** We searched MEDLINE, Embase, Web of Science, Scopus, Cochrane CENTRAL, and Google Scholar for studies diagnosing CE via CD138 immunostaining, assessing microbiota with molecular techniques. Data extraction, quality assessment, and meta-analysis were performed. **Results:** Twenty-two studies including 4022 women were analyzed. CE was associated with reduced prevalence of *Lactobacillus*-dominated microbiota and increased detection of non-*Lactobacillus* species, particularly *Streptococcus* spp., *Enterococcus* spp., *Escherichia coli*, *Staphylococcus* spp., *Ureaplasma* spp., and *Gardnerella vaginalis*. In the meta-analysis (2947 women), *Enterococcus* spp. and *Ureaplasma* spp. were significantly more prevalent in women with CE, whereas *Streptococcus* spp., *E. coli*, *Staphylococcus* spp. and *G. vaginalis* showed non-significant trends. Only *E. coli* and *Streptococcus* spp. showed significant heterogeneity between-studies. **Conclusions:** CE is linked to microbial dysbiosis with reduced *Lactobacillus* dominance and enrichment of potentially pathogenic taxa, notably *Enterococcus* and *Ureaplasma* spp. These findings suggest that the endometrial microbiome contributes to chronic inflammation and adverse reproductive outcomes, yet heterogeneity and limited evidence call for standardized diagnostics and robust trials before clinical implementation.

## 1. Introduction

Chronic endometritis (CE) is a subtle yet clinically significant pathological condition, defined histologically by the infiltration of endometrial stromal tissue with CD138^+^ plasma cells, indicating a persistent inflammatory response [1,2,3]. While CE often presents asymptomatically, it may manifest through non-specific signs such as abnormal uterine bleeding, pelvic pain, dyspareunia, or chronic leukorrhea [4,5]. Its oligosymptomatic nature contributes to a substantial underdiagnosis in clinical practice, with reported prevalence ranging from 2% to 46% depending on the population examined, diagnostic criteria employed, and methods of detection [6,7].

Over the past decade, the clinical relevance of CE has gained increasing recognition, particularly in the field of reproductive medicine. A growing body of evidence supports a strong association between CE and adverse reproductive outcomes, including unexplained infertility, recurrent implantation failure (RIF), and recurrent pregnancy loss (RPL) [8]. Several studies have identified a higher prevalence of CE in women with unexplained infertility, recurrent implantation failure (RIF), and recurrent pregnancy loss (RPL). It has been demonstrated that targeted antibiotic treatment of CE can significantly improve endometrial receptivity and increase clinical pregnancy rates in women undergoing assisted reproductive technologies (ART). Importantly, several interventional studies have demonstrated that targeted antibiotic therapy can restore endometrial receptivity and significantly improve clinical pregnancy and live birth rates in women undergoing ART [9,10,11]. These findings underscore the therapeutic relevance of identifying and treating CE prior to embryo transfer. Current management strategies primarily rely on empiric or culture-guided antibiotic regimens, most commonly doxycycline-based protocols, with second-line therapies tailored according to microbiological findings [10]. However, therapeutic responses are heterogeneous, recurrence rates are not negligible, and standardized treatment protocols remain lacking [1,4]. Moreover, the absence of internationally accepted diagnostic thresholds, variability in hysteroscopic criteria, and inconsistencies in immunohistochemical assessment further complicate clinical decision-making and limit the integration of CE screening into routine fertility workups [12].

Concurrently, the endometrial microbiome has emerged as a novel and promising factor in reproductive medicine [13]. Historically considered a sterile environment, the uterine cavity is now recognized to host a unique microbiota, as revealed by advances in high-throughput sequencing techniques such as 16S rRNA gene sequencing [14,15]. In healthy conditions, this microbial ecosystem is typically characterized by a predominance of *Lactobacillus* spp., which contribute to the maintenance of epithelial barrier integrity, modulation of local immune responses, and protection against colonization by potentially pathogenic microorganisms [16]. However, variations in the composition of the endometrial microbiota have been increasingly reported, with some women exhibiting non-*Lactobacillus*-dominated microbial profiles. Such alterations have been associated with several gynecological conditions, including bacterial vaginosis, pelvic inflammatory disease, and CE [17].

Mounting evidence suggests that the composition of the endometrial microbiome may play a pivotal role in modulating endometrial immune responses and, consequently, reproductive outcomes [18]. Accumulating evidence indicates that these microbial shifts may promote endometrial inflammation through multiple virulence and host–microbe interaction mechanisms. Dysbiotic communities enriched with anaerobic bacteria—such as *Gardnerella vaginalis*, *Atopobium vaginae*, *Prevotella* spp., and *Bacteroides* spp.—may contribute to the disruption of epithelial barriers, biofilm formation, and activation of pro-inflammatory signaling pathways within the endometrium [19]. These processes can stimulate the recruitment of immune cells, the release of inflammatory cytokines, and the development of the chronic inflammatory environment characteristic of CE. Such inflammatory alterations may compromise endometrial receptivity and impair embryo implantation, which relies on a finely regulated interaction between a developmentally competent embryo and a receptive endometrium during the “window of implantation” [20,21]. Disruptions in this immunological and molecular synchrony may negatively influence embryo adhesion and early trophoblast invasion [22].

Given the potential role of endometrial dysbiosis in CE and reproductive failure, increasing attention has been directed toward strategies aimed at restoring microbial balance within the uterine environment. Current approaches include antibiotic therapy targeting CE-associated pathogens, as well as emerging strategies such as probiotic supplementation and microbiome-modulating interventions designed to re-establish *Lactobacillus*-dominant microbial communities. Although these approaches remain under active investigation, they highlight the growing recognition of the endometrial microbiome as a potentially modifiable factor in reproductive health.

Nevertheless, the causal directionality and mechanistic underpinnings of the CE–microbiome relationship remain unclear. Whether microbial dysbiosis precedes and contributes to CE, or whether chronic inflammation alters the microbial niche, is still under investigation. The aim of this systematic review is to evaluate the association between CE and the endometrial microbiome, with particular emphasis on the pathophysiological mechanisms involved and their clinical relevance.

## 2. Materials and Methods

### 2.1. Registration of Protocols

**Figure 1:**
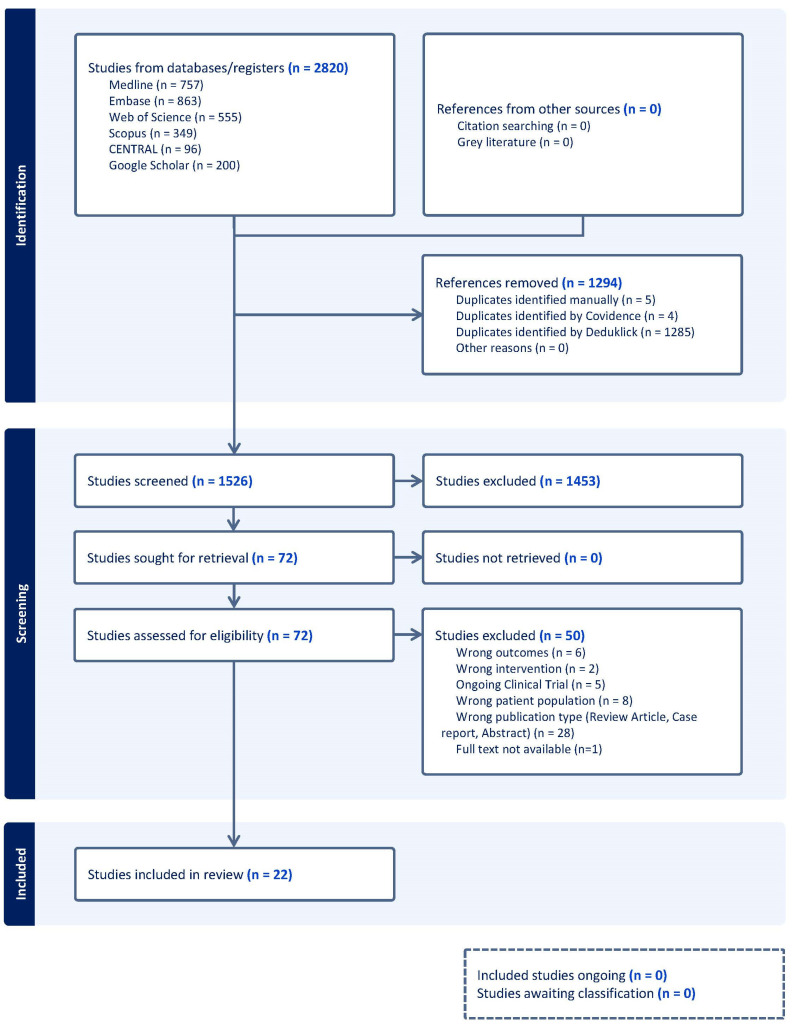
PRISMA flow chart. Flowchart of the bibliography search and selection process.

The protocol was registered in the Prospective International Registry of Systematic Reviews, PROSPERO (registered number CRD420251107580) (Figure 1). The guidelines for the Preferred Reporting Items for Systematic Reviews and Meta-Analyses (PRISMA) have been used [23].

### 2.2. Search Strategy

To identify potentially relevant publications on the topic, two main search concepts were built: (1) chronic endometritis and (2) endometrial microbiome. A search strategy was designed and investigated in MEDLINE, Embase, Web of Science, Scopus, Cochrane Central Register of Controlled Trials (CENTRAL), and Google Scholar. An experienced medical information specialist (TK) initially constructed the search strategy in Embase and validated it against a predefined set of key references to ensure the inclusion of relevant publications. Following refinement, the strategy was tailored to each database using a combination of database-specific controlled vocabulary and free-text terms. The free-text search incorporated synonyms, acronyms, and related terms. Studies conducted exclusively in animals were excluded using an index-term-based filter. In addition, the study types, systematic reviews, meta-analyses, and case reports were excluded as they did not meet the inclusion criteria. The results were limited to publications in English, French, German, and Spanish. The search was finalized on 24 September 2025 (Supplementary File S1). The results were deduplicated using the automated deduplication tool of Deduklick [24]. Screening, data extraction, and study assessment were performed in the screening tool Covidence. The completed PRISMA checklist is provided as Supplementary Materials.

### 2.3. Inclusion and Exclusion Criteria

Studies were independently assessed for inclusion using Covidence software 2.0 (www.covidence.org, accessed on 18 March 2026) [25] and conducted in two sequential stages: (1) title and abstract screening and (2) full-text review. Three investigators (AV, VV, and AK) independently screened all retrieved records. Prior to formal screening, eligibility criteria were predefined and piloted to ensure consistency in their application.

Inclusion criteria were as follows:

- Original research articles (prospective or retrospective observational studies).
- Studies evaluating the association between chronic endometritis (CE) and the endometrial microbiome.
- Diagnosis of CE confirmed by CD138 immunohistochemical staining on endometrial biopsy or uterine specimens.
- Assessment of endometrial microbial composition using molecular diagnostic techniques (e.g., 16S rRNA gene sequencing or other validated molecular platforms).

Exclusion criteria included the following:

- Reviews, case reports, editorials, conference abstracts without full text, and animal studies.
- Studies lacking clear diagnostic criteria for CE or not using CD138 immunostaining.
- Studies evaluating only vaginal or cervical microbiota without specific assessment of the endometrial microbiome.
- Articles with insufficient data to extract relevant outcomes.

### 2.4. Data Extraction

The extracted data were independently summarized and reviewed by two investigators (AV and AK). Primary variables of interest included study population characteristics such as patient age and the presence of CE, as well as the diagnostic methods and parameters used to identify CE, including CD138 immunostaining (Table 1). Additional variables comprised the assessment of the endometrial microbiome (Table 2 and Table 3). After independent extraction, the two datasets were compared for accuracy and completeness. Any discrepancies were discussed and resolved by consensus through re-examination of the original article. If disagreement persisted, a third investigator reviewed the study to reach a final decision.

### 2.5. Quality Assessment

Study quality was assessed using the Newcastle–Ottawa Scale (NOS) [47]. Each study was evaluated across three domains: selection of study groups (0–4 stars), comparability of groups (0–2 stars), and assessment of outcomes/exposure (0–3 stars). Overall study quality was classified as good (3–4 stars in the selection domain and 1–2 stars in the comparability domain and 2–3 stars in the outcome/exposure domain), fair (2 stars in the selection domain and 1–2 stars in the comparability domain and 2–3 stars in the outcome/exposure domain), or poor (0–1 star in the selection domain or 0 stars in the comparability domain or 0–1 star in the outcome/exposure domain). All included studies were independently assessed for risk of bias by two reviewers (AV and MS). Any discrepancies were resolved through consensus. Scoring criteria were applied in accordance with the definitions presented in Table 4.

### 2.6. Data Synthesis

All analyses were conducted using the metafor package in R statistical software (Version 2025.05.1+513). Odds ratios (ORs) with 95% confidence intervals (95% CIs) were calculated for each study to compare the prevalence of specific microbial species in women with chronic endometritis versus controls. Log-transformed ORs were pooled using both fixed-effect (inverse-variance) and random-effects models, with the latter fitted using restricted maximum likelihood (REML). In the presence of relevant heterogeneity, random-effects estimates were considered primary. Between-study heterogeneity was assessed using Cochran’s Q test (*p* < 0.10), the I^2^ statistic, and the between-study variance (τ^2^). Sensitivity analyses were performed using a leave-one-out approach and influence diagnostics to evaluate the robustness of pooled estimates. When at least ten studies were available, random-effects meta-regression analyses were conducted to explore potential sources of heterogeneity, including study design, diagnostic criteria, microbiological assessment method, and geographic region. Publication bias and small-study effects were assessed through visual inspection of funnel plots; when asymmetry was detected, the trim-and-fill method was applied. All analyses were performed separately for each microbial species, and study weights were reported for both models.

## 3. Results

### 3.1. Results of the Systematic Review

**Figure 2:**
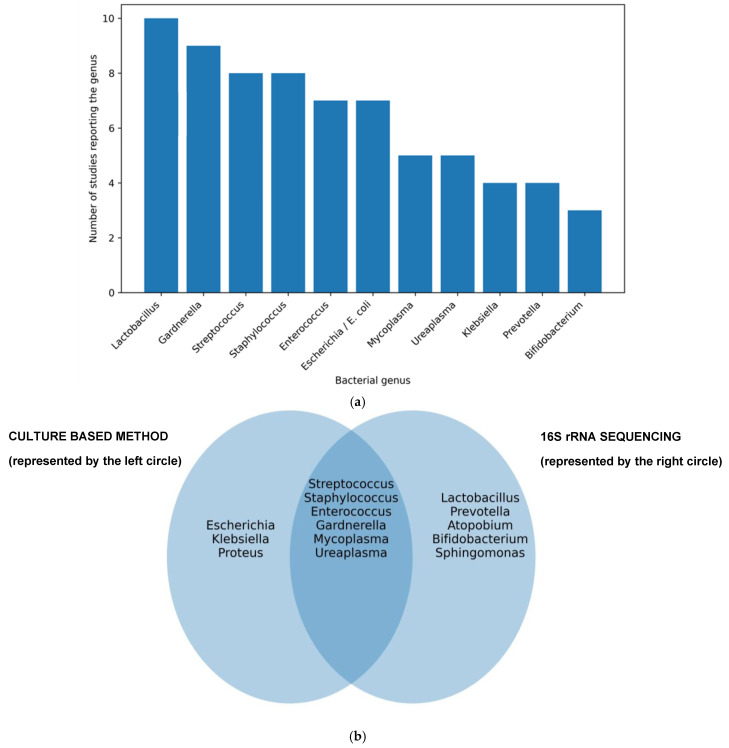
(**a**) Most frequently reported bacterial genera associated with chronic endometritis across studies published between 2006 and 2025. The bar plot illustrates the number of studies reporting each bacterial genus identified in the endometrial microbiome of patients with chronic endometritis. (**b**) Venn diagram illustrating bacterial genera reported in chronic endometritis studies according to the detection method used. Genera identified by culture-based methods are shown in the left circle, whereas those identified by 16S rRNA sequencing or PCR-based methods are shown in the right circle. The overlapping area represents genera detected by both approaches.

A total of 2820 records were retrieved through the database search. Following duplicate removal, 1526 abstracts were screened, and 72 articles were selected for full-text evaluation. Of these, 22 studies fulfilled the inclusion criteria and were included in the systematic review (Figure 2a), encompassing 4022 women. Studies were most commonly excluded because of missing or irrelevant outcome measures, inappropriate study design, or an ineligible study population.

Microbiota composition in women with chronic endometritis (Figure 2b): Overall, women with CE showed a lower prevalence of *Lactobacillus*-dominant endometrial microbiota compared to women without CE [29,31]. Although reported prevalence varied, a clearly significantly lower relative abundance was described in CE repeatedly (CE 40.88% vs. non-CE 64.22%, *p* < 0.05) [46] and up to a 42.7-fold decrease (CE 1.89% vs. non-CE 80.7%) [30]. One specific study suggested a species-specific shift with a higher relative dominance of *Lactobacillus* iners in women with CE rather than uniform depletion [39].

Distribution of non-*Lactobacillus* bacteria in chronic endometritis: Non-*Lactobacillus* taxa were more frequently detected in women with CE, particularly *Gardnerella vaginalis*, *Ureaplasma* spp., and *Prevotella* spp. [30,39,44]. However, these microorganisms were also identified in women without histological evidence of CE, limiting their specificity as diagnostic markers [32,36,45].

Alterations in endometrial microbiota were additionally explored in specific reproductive subgroups, including women with recurrent pregnancy loss, recurrent miscarriage, and recurrent implantation failure, although results remained heterogeneous [30,39,45]. Concordance between vaginal, cervical, and endometrial microbiota was poor, indicating that lower genital tract sampling does not reliably predict endometrial microbial composition [27,35].

Substantial methodological heterogeneity was observed across studies, both in microbiota assessment—ranging from culture-based techniques to real-time PCR and next-generation sequencing [32,36,38]—and in CE diagnosis, which was primarily based on CD138-positive plasma cell counts using variable cut-off thresholds or, in some studies, hysteroscopic criteria [27,28,33,42,44,46].

### 3.2. Results of the Meta-Analysis

Of the 22 included studies comprising a total of 4022 women, only 2947 women were eligible for inclusion in the meta-analysis because they provided comparable quantitative data on the presence of specific microorganisms. The remaining studies reported only binomial data (presence/absence), which could not be pooled quantitatively and were therefore excluded from the meta-analysis according to the predefined exclusion criteria.

**Figure 3:**
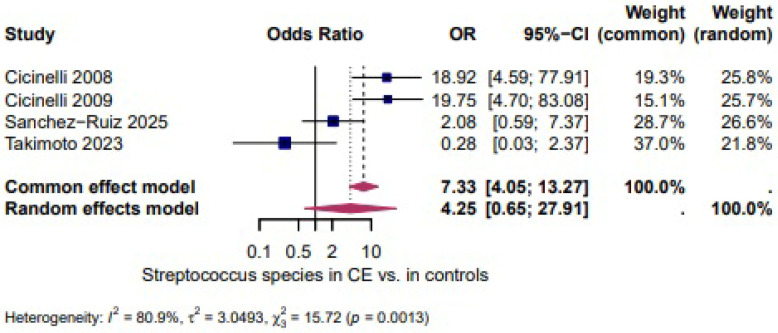
*Streptococcus* spp. in CE vs. Controls [27,28,39,46]. Forest plot of the odds ratio (OR) for the presence of *Streptococcus* spp. in patients with chronic endometritis compared with controls. Each dark blue square represents the study-specific OR, with the size of the square proportional to the study weight, and the horizontal lines indicate the 95% confidence intervals (CI). The red rhombus represents the pooled OR and its 95% CI derived from the fixed-effect (common effect) and random-effects models. OR values greater than 1 indicate a higher prevalence of *Enterococcus species* in chronic endometritis compared with controls.

*Streptococcocus species* (spp.): Four studies [27,28,39,46] evaluated the presence of *Streptococcocus* spp. and demonstrated a higher, but not statistically significant, likelihood of *Streptococcocus* spp. in CE (OR = 4.25, 95% CI: 0.65–27.91). The heterogeneity test revealed significant heterogeneity (I^2^ = 80.9%, τ^2^ = 3.0493, χ^2^ = 15.72, *p* = 0.0013). (Figure 3).

**Figure 4:**
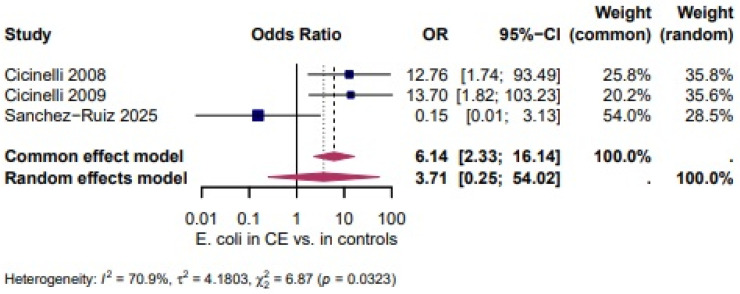
*E. Coli* in CE vs. Controls. [27,28,46] For details, see legend of Figure 3.

*Escherichia coli* (*E. coli*): Three studies [27,28,46] evaluated the presence of *E. coli* and showed a higher, but not statistically significant, likelihood of *E. coli* in CE (OR = 3.71, 95% CI: 0.25–54.02). The heterogeneity test revealed significant heterogeneity (I^2^ = 70.9%, τ^2^ = 4.1803, χ^2^ = 6.87, *p* = 0.0323). (Figure 4).

**Figure 5:**
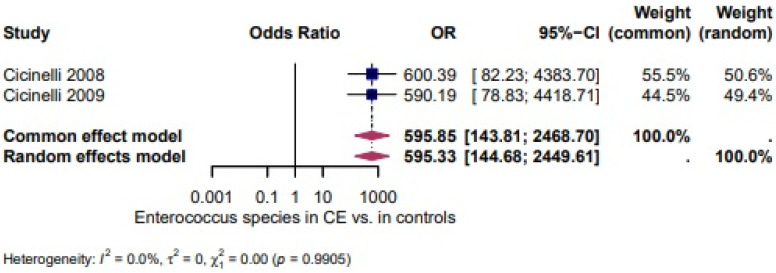
*Enterococcus* spp. in CE vs. Controls [27,28]. For details, see legend of Figure 3.

*Enterococcus* spp.: Two studies [27,28] evaluated the presence of *Enterococcus* spp. and revealed very high odds in CE (OR = 595.33 95% CI: 1.44–2449). There was no heterogeneity between the studies (I^2^ = 0.0%, τ^2^ = 0, χ^2^ = 35.26, *p* = 0.9905) (Figure 5).

**Figure 6:**
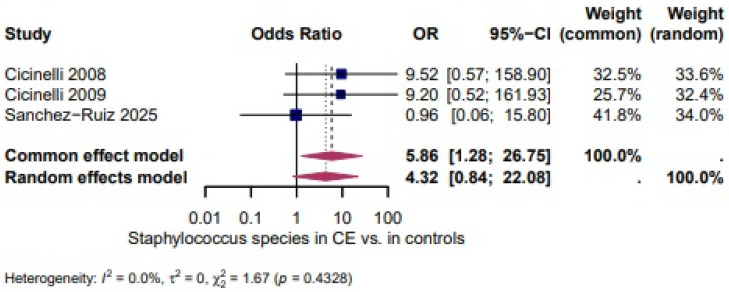
*Staphylococcus* spp. in CE vs. Controls [27,28,46]. For details, see legend of Figure 3.

*Staphylococcus* spp: A higher, but not statistically significant, odds of *Staphylococcus* spp. in CE was shown in three studies [27,28,46] (OR = 4.32, 95% CI: 0.84–22.08). There was no heterogeneity between the studies (I^2^ = 0.0%, τ^2^ = 0, χ^2^ = 1.67, *p*= 0.4328) (Figure 6).

**Figure 7:**
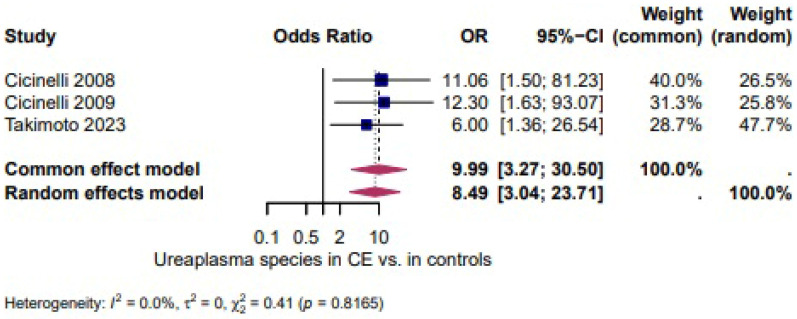
*Ureaplasma* spp. in CE vs. Controls [27,28,39]. For details, see legend of Figure 3.

*Ureaplasma* spp.: *Ureaplasma* spp. appeared more often in CE [27,28,39] (OR = 8.49, 95% CI: 3.04–23.71). The heterogeneity test revealed no heterogeneity (I^2^ = 0.0%, τ^2^ = 0, χ^2^ = 0.41, *p*= 0.8165) (Figure 7).

**Figure 8:**
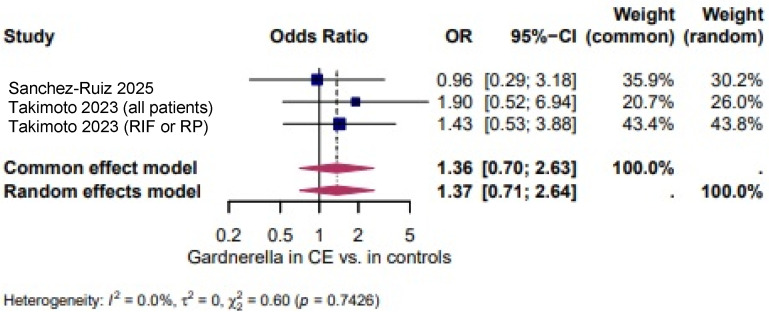
*Gardnerella vaginalis* in CE vs. Controls [39,46]. For details, see legend of Figure 3.

*Gardnerella vaginalis*: The odds of *Gardnerella vaginalis* in CE was comparable to controls [39,46] (OR = 1.37, 95% CI: 0.71–2.64). The heterogeneity test revealed non-significant heterogeneity (I^2^ = 0.0%, τ^2^ = 0, χ^2^ = 0.60, *p* = 0.7426). (Figure 8).

**Figure 9:**
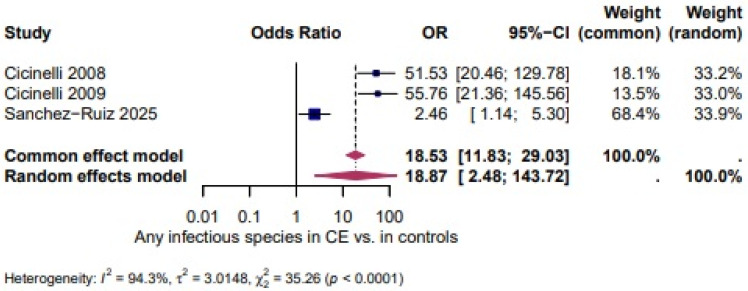
Infectious Species in CE vs. Controls [27,28,46]. For details, see legend of Figure 3.

Any infectious species: Three studies [27,28,46] evaluated the presence of any infectious species in women with chronic CE. There was a significantly higher odds in CE (OR = 18.87, 95% CI: 2.48–143.72), with high heterogeneity between the studies, The heterogeneity test revealed significant heterogeneity (I^2^ = 94.3%, τ^2^ = 3.0148, χ^2^ = 35.26, *p* < 0.0001). (Figure 9).

In the quantitative synthesis including 2947 women, *Enterococcus* spp. and *Ureaplasma* spp. were significantly more prevalent in women with CE than in controls, whereas *Streptococcus* spp., *Escherichia coli*, *Staphylococcus* spp. and *Gardnerella vaginalis* showed non-significant trends with substantial variability across studies. Overall, the odds of detecting any infectious species were also increased in CE, but this composite endpoint was characterized by very high between-study heterogeneity and wide confidence intervals [1].

Four studies evaluated the presence of *Streptococcus* spp. and demonstrated a higher, but not statistically significant, likelihood of *Streptococcus* spp. in CE (OR = 4.25, 95% CI 0.65–27.91; I^2^ = 80.9%, τ^2^ = 3.0493, χ^2^ = 15.72, *p* = 0.0013; Figure 3). Three studies assessed *E. coli* and showed a higher, but not statistically significant, likelihood of *E. coli* in CE (OR = 3.71, 95% CI 0.25–54.02; I^2^ = 70.9%, τ^2^ = 4.1803, χ^2^ = 6.87, *p* = 0.0323; Figure 4). Two studies evaluated *Enterococcus* spp. and revealed very high odds in CE (OR = 595.33, 95% CI 1.44–2449) with no heterogeneity between studies (I^2^ = 0.0%, τ^2^ = 0, χ^2^ = 0.01, *p* = 0.9905; Figure 5) [1].

A higher, but not statistically significant, odds of *Staphylococcus* spp. in CE was shown in three studies (OR = 4.32, 95% CI 0.84–22.08; I^2^ = 0.0%, τ^2^ = 0, χ^2^ = 1.67, *p* = 0.4328; Figure 6). *Ureaplasma* spp. appeared more often in CE (OR = 8.49, 95% CI 3.04–23.71; I^2^ = 0.0%, τ^2^ = 0, χ^2^ = 0.41, *p* = 0.8165; Figure 7). The odds of *Gardnerella vaginalis* in CE were comparable to controls (OR = 1.37, 95% CI 0.71–2.64; I^2^ = 0.0%, τ^2^ = 0, χ^2^ = 0.60, *p* = 0.7426; Figure 8). Three studies evaluated the presence of any infectious species in women with CE and showed significantly higher odds (OR = 18.87, 95% CI 2.48–143.72; I^2^ = 94.3%, τ^2^ = 3.0148, χ^2^ = 35.26, *p* < 0.0001; Figure 9), although the extreme heterogeneity and wide confidence intervals limit the robustness of this composite outcome.

## 4. Discussion

The aim of this systematic review and meta-analysis was to evaluate the association between CE and alterations in the endometrial microbiome. To our knowledge, this is the first meta-analysis to investigate the presence of potentially pathogenic microorganisms in women with histologically confirmed CE compared to controls.

Our qualitative synthesis consistently showed that CE was associated with a reduction in *Lactobacillus*-dominated microbiota and a higher detection of non-*Lactobacillus* taxa. The quantitative meta-analysis further indicated that *Enterococcus* spp. and *Ureaplasma* spp. were significantly more prevalent in women with CE. In contrast, *Streptococcus* spp., *Escherichia coli*, *Staphylococcus* spp., and *Gardnerella vaginalis* showed non-significant trends toward higher odds in CE. Among the evaluated taxa, the strongest association was observed for *Enterococcus* spp.; however, the very wide confidence intervals and the small number of contributing studies indicate considerable statistical uncertainty. These estimates should therefore be interpreted cautiously and regarded as hypothesis-generating rather than definitive evidence. For *Streptococcus* spp. and the composite category “any infectious species,” the direction of effect similarly suggested a higher prevalence in CE, although these associations were weakened by substantial between-study heterogeneity. Given the presence of considerable heterogeneity across several outcomes, interpretation of pooled estimates primarily relied on random-effects models, while fixed-effect estimates were considered with caution because they may overestimate the precision of the pooled effects.

From a biological perspective, these findings support the hypothesis that CE is associated with alterations in the endometrial microbial environment rather than the presence of a single pathogenic organism. CE has traditionally been described as a chronic, low-grade inflammatory condition of the endometrium often linked to ascending genital tract infections [16,39]. However, accumulating evidence suggests that it may instead reflect a broader state of endometrial microbiota dysbiosis. In this context, the observed reduction in *Lactobacillus*-dominated communities—combined with a higher detection of taxa such as *Enterococcus*, *Streptococcus*, *Escherichia coli*, and *Gardnerella vaginalis*—may contribute to the maintenance of a persistent inflammatory environment within the endometrium. Notably, however, our results do not support the use of individual microorganisms as specific microbiological markers of CE [18]. Our findings support this perspective, showing a clear association between CE and the presence of these microorganisms, further reinforcing the link between microbial dysbiosis and endometrial inflammation. However, although the pooled odds for any infectious species were markedly elevated in women with CE, the very wide confidence intervals and extremely high heterogeneity indicate that this composite endpoint is unlikely to serve as a robust diagnostic marker in clinical practice. Similarly, while *Gardnerella vaginalis* was frequently detected in both CE and non-CE samples, our meta-analysis does not support its use as a specific microbiological marker for CE. Rather than focusing on single species, future research should aim to define characteristic community-level patterns and functional signatures that distinguish health, dysbiosis and CE-related inflammation.

CE is increasingly recognized not merely as a localized endometrial pathology but as a condition that disrupts the complex immuno-microbial equilibrium essential for reproductive success [4,6,7,48]. The chronic inflammatory state associated with CE has been linked to increased levels of pro-inflammatory cytokines, including interleukin-6 (IL-6) and tumor necrosis factor-α (TNF-α), as well as alterations in Th1/Th2 immune responses. Such immunological changes may impair key endometrial processes, including stromal remodeling, angiogenesis, and decidualization [4,6,49]. Parallel to these immunological disturbances, a growing body of evidence implicates endometrial microbiota dysbiosis in implantation failure and early pregnancy loss [9,39,50]. In particular, the depletion of *Lactobacillus* spp. and overrepresentation of potentially pathogenic taxa have been correlated with reduced implantation rates, recurrent implantation failure (RIF), and increased miscarriage risk [12,48,51,52]. CE may thus act as a pathological interface between microbial imbalance and defective immune signaling, disrupting the cytokine and chemokine networks and altering the activation state of local immune cells [53]. Such dysregulation compromises embryo–endometrial synchrony during the window of implantation, thereby contributing to unexplained infertility and poor reproductive outcomes [4,48].

Recent studies have increasingly emphasized the importance of genital microbiota composition in reproductive outcomes, particularly regarding the role of microbial dysbiosis in female infertility [18]. Alterations in the vaginal and endometrial microbiota, especially a reduction in *Lactobacillus* spp. and overrepresentation of anaerobic bacteria, have been associated with impaired implantation, RIF, and RPL. In a comprehensive analysis [19], Baud et al. (2025) identified distinct genital microbial profiles in infertile couples, implicating dysbiosis as a shared contributing factor to reproductive dysfunction in both partners [19], frequently observed in women with RIF and RPL, may represent a clinical bridge between microbiota imbalance and endometrial dysfunction. Consistent with this perspective, our systematic review included 22 studies investigating the relationship between genital microbiota, CE, and infertility, reflecting the growing interest in the microbiome as a potentially modifiable factor in reproductive medicine.

Although empirical antibiotic therapy remains the standard approach for treating CE, its therapeutic limitations are increasingly recognized [54]. Importantly, the resolution of CE following antibiotic treatment does not necessarily indicate the re-establishment of a healthy microbial environment. In some cases, they may even aggravate dysbiosis by disrupting commensal populations, particularly *Lactobacillus* spp., which are essential for maintaining mucosal immune homeostasis [9]. These limitations highlight the need for more integrative treatment strategies that go beyond microbial eradication and focus on promoting microbial recovery. Adjunctive use of probiotics—especially strains such as *Lactobacillus crispatus*, *L. jensenii*, or *L. gasseri*—as well as prebiotics or synbiotics, has been proposed as a means to reestablish eubiosis and modulate immune responses [55,56]. However, robust clinical evidence supporting these approaches in the context of CE remains limited. At present, evidence for microbiota-targeted interventions in CE remains restricted to small pilot studies and extrapolation from vaginal microbiota research, and no randomized controlled trials have yet demonstrated that probiotic or symbiotic strategies improve live birth rates in women with CE [55,57]. In particular, strategies aimed at reintroducing or sustaining *Lactobacillus* dominance within the endometrial niche may be crucial for restoring endometrial receptivity and improving fertility outcomes in women with microbiota-associated reproductive disorders.

A central challenge in the diagnosis of CE lies in the discordance between histopathological marker, such as CD138^+^ plasma cell infiltration, and molecular microbiome profiles. Several studies have demonstrated that histological CE can be identified even in the absence of detectable microbial dysbiosis, while significant microbial alterations may occur without classical histological inflammation [5,48,58]. These discrepancies raise important questions regarding the biological nature of CE—specifically, whether it represents a distinct inflammatory pathology or a histological manifestation of a broader microbial–immune disequilibrium. Integrating molecular diagnostic approaches, such as 16S rRNA sequencing and immunological profiling, with conventional histopathological assessment may therefore improve diagnostic accuracy, enable the identification of clinically relevant CE subtypes, and support more personalized therapeutic strategies [14,56].

Furthermore, the association between CE and microbial dysbiosis has been reported in other gynecological conditions, including isthmocele and adenomyosis, suggesting the presence of shared pathophysiological mechanisms involving chronic inflammation, altered tissue remodeling, and microbiota–immune interactions [59]. These observations support the hypothesis that CE may represent a heterogeneous syndrome comprising at least two partially overlapping phenotypes: an immune-dominant, histology-driven form and a microbiota-associated dysbiotic form. Future research should therefore aim to integrate quantitative CD138 scoring with cytokine profiling, immune cell characterization, and high-resolution microbiome analysis to define biologically meaningful CE subtypes. Such an approach may also help explain the variable response to antibiotic treatment observed in clinical practice, where some women experience reproductive improvement following histological resolution of CE, while others continue to experience reproductive failure, potentially due to persistent microbial or immune dysregulation.

The accumulating evidence linking CE and endometrial microbiota dysbiosis with adverse reproductive outcomes, including RIF, RPL, and unexplained infertility, has important clinical implications. In this context, targeted screening for CE and microbial imbalance in high-risk populations appears justified, particularly among women with a history of implantation failure or early pregnancy loss. Nevertheless, routine universal screening is currently limited by a lack of robust evidence supporting its diagnostic accuracy, cost-effectiveness, and clinical utility. The identification and validation of microbial biomarkers indicative of endometrial health may, in the future, enable stratified screening approaches and facilitate more personalized therapeutic strategies. Advancing this field requires longitudinal studies to elucidate the temporal dynamics of the endometrial microbiome in relation to CE onset, progression, and treatment response.

From a clinical perspective, our data argue against universal microbiome-based screening in asymptomatic women and rather support a targeted approach focusing on high-risk groups such as patients with RIF, RPL or unexplained infertility. In these populations, a combined assessment of histological markers of CE and molecular microbiome profiling may help to distinguish predominantly inflammatory, infection-driven phenotypes from dysbiosis-dominated patterns, which might require different therapeutic strategies. While antibiotic regimens remain the standard of care for CE, our findings and previous work suggest that antibiotic monotherapy may insufficiently restore a *Lactobacillus*-dominated microbiota and could even aggravate dysbiosis in some cases. At present, evidence for probiotic or symbiotic interventions in CE is limited to small pilot studies and extrapolation from vaginal microbiota research, and no randomized controlled trials have yet demonstrated an improvement in live birth rates with microbiota-targeted therapies in this setting.

Even though our study strictly followed the recommendations to provide high-quality summary reports of evidence, some limitations are evident: First, the limited number of included studies and participants may have reduced statistical power and the precision of pooled estimates, restricting the strength of subgroup and meta-regression analyses. Second, considerable heterogeneity existed across studies, largely due to variability in diagnostic criteria for chronic endometritis (e.g., differences in CD138 thresholds, sampling methods, and histological interpretation). This lack of standardization may have introduced misclassification bias and influenced between-study variability. Third, most included studies were observational, limiting causal inference and increasing susceptibility to selection bias and residual confounding. Additionally, inconsistencies in microbiota assessment methods, sequencing techniques, and definitions of microbial profiles may have affected comparability and the reliability of pooled microbial associations. These factors should be considered when interpreting the findings and underscore the need for standardized diagnostic and microbiome evaluation protocols in future research.

Furthermore, randomized controlled trials assessing the efficacy of combined treatment regimens, such as antibiotics paired with probiotics or immunomodulatory agents, are essential to define mechanism-based therapies. A critical gap remains in the definition of a “healthy” endometrial microbiome. Establishing normative microbial profiles will be key not only to improve diagnostic precision but also to guide interventions aimed at restoring reproductive competence.

## 5. Conclusions

By quantitatively synthesizing data from 22 heterogeneous studies, our work offers the first pooled estimates for the association between specific microbial taxa and histologically confirmed CE. These results provide a structured framework for hypothesis generation and the design of future mechanistic and interventional studies, but they should not yet be used to define diagnostic cut-offs or to guide microbiome-based interventions in routine clinical practice. Advancing this field will require adequately powered, standardized and longitudinal studies to elucidate the temporal dynamics of the endometrial microbiome in relation to CE onset, progression and treatment response.

## Figures and Tables

**Table 1:** Characteristics of included studies.

Author, Year of Publication	Country	Study Design	Controls	Number of Participants of Interest	Total Number of Participants	Number of Participants in Control Group	Age (y) Mean SD or Median Study Group	Age (y) Mean SD or Median (Control Group)
Andrews, W. et al. 2006 [26]	USA	Observational cohort	NA	820	769	NA	23.5 ± 5.6	NA
Cicinelli, E. et al. 2008 [27]	Italy, Switzerland	Prospective diagnostic	Yes	2190	538	100	35.7 ± 8.2	36.3 ± 8.3
Cicinelli, E. et al. 2009 [28]	Italy, Switzerland	Prospective controlled	Yes	281	181	100	36.1 ± 8.3	36.3 ± 8.3
Cicinelli, E. et al. 2014 [29]	Italy, France	Retrospective	NA	360	208	152	30.5	NA
Liu, Y. et al. 2019 [30]	China	Case–control observational	Yes	130	130	NA	24–45	NA
Voroshilina, E. et al. 2020 [31]	Russia	Prospective observational	Yes	72	72	NA	33 ± 5.2	NA
Chen, P. et al. 2021 [32]	China	Observational cohort	Yes	112	104	NA	NA	NA
Chen, W. et al. 2021 [33]	China	Prospective cohort	NA	94	94	69	30.16	NA
Lozano, F. et al. 2021 [34]	Spain	Prospective cohort	NA	60	54	NA	39.2	NA
Voropaeva, N. et al. 2021 [35]	Russia	Cross-sectional	NA	47	47	NA	35.38 ± 5.19	NA
Moreno, I. et al. 2022 [36]	Multicentre	Multicentre prospective observational cohort	NA	452	342	NA	36 (21–49)	NA
Tanaka, S. et al. 2022 [37]	Japan	Interim analysis case–control	NA	130	123	103	38	NA
Chen, Q. et al. 2023 [14]	China	Observational cohort	NA	71	113	NA	32.11 ± 4.46	NA
Liang, J. et al. 2023 [38]	China	Prospective	Yes	134	134	NA	33 ± 7.6	NA
Takimoto, K. et al. 2023 [39]	Japan	Prospective case–control	Yes	80	80	29	RIF/ RPL/ controls reported	NA
Han, Y. et al. 2024 [40]	China	Cross-sectional	Yes	114	98	49	33.5 ± 3.8	33.7 ± 3.8
Hmaidan S. et al. 2024 [41]	USA	Retrospective cohort	NA	410	400	NA	NA	NA
Kapetanios, V. et al. 2024 [42]	Greece	Prospective cohort	NA	287	194	NA	37.36 ± 4.79	37.32 ± 3.87
Nandagopal, M. et al. 2024 [43]	India	Retrospective diagnostic accuracy	Yes	318	318	NA	18–50	NA
Zhang, H. et al. 2024 [44]	China	Prospective observational	NA	83	80	40	30.57 ± 2.64	32.88 ± 2.03
Klimaszyk, K. et al. 2025 [45]	Poland, Denmark	Cross-sectional observational	NA	98	90	NA	32 ± 4.54	33 ± 5.96
Sanchez–Ruiz, R. et al. 2025 [46]	Spain	Cross-sectional retrospective descriptive	Yes	110	110	NA	35.1 ± 4.24	35.33 ± 3.83

Overview of cohort studies Abbreviations: N/A (not available); PC (plasma cells); RIF (recurrent implantation failure); RPL (recurrent pregnancy loss).

**Table 2:** CE-/Microbiome-associated conditions.

Author, Year of Publication	Infertility Associated	Type of Infertility Patient	Diagnosis Method of CE	Methodology	CE Definition	Predominant Microbiome
Andrews, W. et al. 2006 [26]	No	NA	Endometrial biopsy	Immunohistochemistry	CD138 cell count	39.1% any anaerobe; 14.9% anaerobic *Gram-neg bacilli*; 13.8% anaerobic *Gram-positive cocci*; 13.8% *M. hominis*; 71.6% *G. vaginalis*; 4.2% *Mobiluncus* spp.
Cicinelli, E. et al. 2008 [27]	Yes	Bleeding, infertility, myoma	Hysteroscopy	Immunohistochemistry	Hysteroscopic criteria	*Escherichia coli*, *Streptococcus*, *Staphylococcus*, *Enterococcus faecalis*, *Chlamydia*, *Ureaplasma*, *yeast*
Cicinelli, E. et al. 2009 [28]	No	Partially	Hysteroscopy	Immunohistochemistry	Hysteroscopic criteria	*Corynebacterium*, *Enterococcus faecalis*, *E. coli*, *Gardnerella vaginalis*, *Klebsiella pneumoniae*, *Proteus* spp., *Pseudomonas aeruginosa*, *Candida*, *Chlamydia*, *Ureaplasma*
Cicinelli, E. et al. 2014 [29]	Yes	Recurrent miscarriage	Mini-hysteroscopy, culture	Immunohistochemistry	Hysteroscopic criteria and biopsy	*Staphylococcus* spp., *Enterococcus faecalis*, *Streptococcus* spp., *E. coli*, *Candida*, *Klebsiella*, *Mycoplasma/Ureaplasma*, *Chlamydia*
Liu, Y. et al. 2019 [30]	Yes	Infertility or recurrent miscarriage	Endometrial biopsy and uterine lavage, 16S rRNA sequencing	Immunohistochemistry	>5.15/10 mm^2^ plasma cells	*Lactobacillus*, *Enterococcus faecalis*, *Staphylococcus*, *Gardnerella*, *Mycoplasma*, *Ureaplasma*, *Chlamydia*
Voroshilina, E. et al. 2020 [31]	Yes	Infertility, abortion history, CE	Endometrial biopsy	Immunohistochemistry and Real Time PCR DNA extraction	NA	24 bacterial groups incl. *Lactobacillus*, *Staphylococcus*, *Streptococcus*, *Gardnerella*, *Ureaplasma*, *Mycoplasma*, *Chlamydia*
Chen, P. et al. 2021 [32]	Yes	RIF cohort with FET	Endometrial transcriptome analysis by 16S rRNA sequencing	Immunohistochemistry	CD138 cell count	Composition of endometrial microorganisms differed between CE and non-CE; *Phyllobacterium* and *Sphingomonas* associated with immune regulation
Chen, W. et al. 2021 [33]	Yes	Recipients of 1 IVF cycle	Hysteroscopy	Immunohistochemistry	>5–18/10 mm^2^ plasma cells	*Firmicutes*, *Actinobacteria*, *Fusobacteria*, *Bacteroidetes*, *Acidobacteria*, *Lactobacillus*, *Halomonas*, *Gardnerella*
Lozano, F. et al. 2021 [34]	Yes	Undergoing ART	Hysteroscopy	Immunohistochemistry	CD138 cell count	*Anaerobacillus*, *Burkholderia*, *Delftia*, *Dialister*, *Lactobacillus*, *Gardnerella*, *Streptococcus*
Voropaeva, N. et al. 2021 [35]	No	NA	Endometrial biopsy	Immunohistochemistry	NA	*Lactobacillus* spp., *Enterobacteriaceae*, *E. coli*, *Klebsiella*, *Staphylococcus*, *Enterococcus*, *Streptococcus*, *Candida*
Moreno, I. et al. 2022 [36]	Yes	Undergoing ART	Endometrial biopsy	Immunohistochemistry. DNA extraction and 16S rRNA sequencing	CD138 cell count	*Atopobium*, *Bifidobacterium*, *Chryseobacterium*, *Gardnerella*, *Haemophilus*, *Klebsiella*, *Neisseria*, *Staphylococcus*, *Streptococcus*
Tanaka, S. et al. 2022 [37]	Yes	Undergoing ART	Hysteroscopy and biopsy	Immunohistochemistry	CD138 cell count	*Rhodanobacter*, *Atopobium*, *Bifidobacterium*, *Streptococcus*, *Enterococcus*, *Staphylococcus*
Chen, Q. et al. 2023 [14]	Yes	IVF-ET failure, recurrent abortion, uterine malformation	Uterine flushing fluid sampling	Immunohistochemistry. DNA extraction and 16S rRNA sequencing	>4/10 mm^2^ plasma cells	*Lactobacillus* 40.88%; *Pseudomonas* 8.10%; *Methylobacterium-Methylorubrum* 8.06%; *Staphylococcus* 5.12%; *Bradyrhizobium* 4.12%; others
Liang, J. et al. 2023 [38]	Yes	NA	Hysteroscopy, CTAB DNA extraction	Immunohistochemistry. DNA extraction and 16S rRNA sequencing	CD138 cell count or polyps	*Firmicutes*, *Actinobacteriota*, *Proteobacteria*, *Fusobacteriota*, *Lactobacillus*, *Staphylococcus*, *Gardnerella*, *Streptococcus*
Takimoto, K. et al. 2023 [39]	Yes	RPL, RIF	Endometrial biopsy	Immunohistochemistry	>5.15/10 mm^2^ plasma cells	*Lactobacillus*, *Ureaplasma*, *Mycoplasma*, *Gardnerella*, *Prevotella*, *Streptococcus*
Han, Y. et al. 2024 [40]	Yes	Endometriosis, tubal factor, anovulation, unexplained	Endometrial biopsy	Immunohistochemistry	>5.15/10 mm^2^ plasma cells	*Bifidobacterium*, *Prevotella*, *Gardnerella*
Hmaidan S. et al. 2024 [41]	Yes	Infertility or RPL	Biopsy	Immunohistochemistry	≥5 plasma cells	*Streptococcus agalactiae*, *Enterococcus faecalis*, *Gardnerella vaginalis*, *E. coli*, *Staphylococcus aureus*
Kapetanios, V. et al. 2024 [42]	Yes	RPL	Hysteroscopy and/or biopsy	Immunohistochemistry	CD138 cell count	Gram-positive 58.75% (*Enterococcus*, *Staphylococcus*); Gram-negative 38.75% (*E. coli*, *Proteus*, *Enterobacter*)
Nandagopal, M. et al. 2024 [43]	Yes	Bleeding, IVF failure, RIF, RPL	Endometrial biopsy	Immunohistochemistry	CD138 cell count	*Enterococcus faecalis*, *E. coli*, *Staphylococcus aureus*, *Mycoplasma* spp., *Streptococcus agalactiae*, *Ureaplasma*, *Mycobacterium*, *Chlamydia*
Zhang, H. et al. 2024 [44]	Yes	RIF	Endometrial biopsy	Immunohistochemistry	>4/10 mm^2^ plasma cells	*Proteobacteria*, *Aminicenantales*, *Chloroflexaceae*, *Lactobacillus*, *Acinetobacter*, *Gardnerella*
Klimaszyk, K. et al. 2025 [45]	Yes	RPL	Diagnostic hysteroscopy and biopsy (Pipelle)	Immunohistochemistry	CD138 cell count	*Chlamydia*, *Enterobacteriaceae*, *Enterococcus*, *E. coli*, *Gardnerella*, *Klebsiella*, *Mycoplasma*, *Neisseria*, *Staphylococcus*, *Streptococcus*
Sanchez–Ruiz, R. et al. 2025 [46]	Yes	Undergoing ART	Endometrial biopsy or hysteroscopy	Immunohistochemistry	>5 plasma cells	*Gardnerella*, *Streptococcus*, *Corynebacterium*, *E. coli*, *Bifidobacterium*, *Prevotella*, *Staphylococcus*

Abbreviations: N/A (not available), ART (assisted reproductive treatment), RIF (recurrent implantation failure), RPL (recurrent pregnancy loss), CE (chronic endometritis), IVF (in vitro fertilization), ET (embryo transfer), FET (frozen embryo transfer), and CD138 (syndecan-1, a transmembrane heparan sulfate proteoglycan predominantly expressed on plasma cells).

**Table 3:** Microbiome detected in each study.

Author, Year of Publication	*Lactobacillus*	*Staphylococcus*	*Streptococcus*	*Enterococcus faecalis*, *E. coli* or *Escherichia*	*Ureaplasma urealyticum* or *Mycoplasma*	*Chlamydia*	*Gardnerella vaginalis*	Chronic Pain/Pelvic Pain Associated	Antibiotic Therapy (Yes/No, Which?)	All Microorganisms Detected
Andrews, W. et al. 2006 [26]	No	No	No	No	No	No	No	NA	NA	*M hominis*, *G*. *vaginalis*, *Mobiluncus* spp.
Cicinelli, E. et al. 2008 [27]	No	Yes	Yes	Yes	Yes	Yes	No	NA	NA	*Escherichia coli*, *Streptococci*, *Staphylococci*, *Enterococcus faecalis*, *Chlamydia*, *Ureaplasma*, *Yeast*
Cicinelli, E. et al. 2009 [28]	No	Yes	Yes	Yes	Yes	Yes	Yes	NA	NA	*Corynebacterium*, *Enterococcus faecalis*, *E. coli*, *Gardnerella vaginalis*, *Klebsiella pneumoniae*, *Proteus* spp., *Pseudomonas aeruginosa*, *Candida*, *Chlamydia*, *Ureaplasma*
Cicinelli, E. et al. 2014 [29]	No	Yes	Yes	Yes	Yes	Yes	No	NA	Yes	*Staphylococcus* spp. (epidermidis, aureus, hemolyticum), *Enterococcus faecalis*, *Streptococcus* spp. (bovis, viridans, agalactiae, itis, milleri), *E. coli*, *Candida*, *Klebsiella pneumoniae*, *Mycoplasma/Ureaplasma*, *Chlamydia*
Liu, Y. et al. 2019 [30]	Yes	Yes	Yes	Yes	Yes	No	Yes	NA	NA	*Lactobacillus*, *Atopobium*, *Bifidobacterium*, *Escherichia-Shigella*, *Prevotella*, *Stenotrophomonas*, *Streptococcus*, *Enterococcus faecalis*, *Staphylococcus*, *Gardnerella*, *Mycoplasma*, *Ureaplasma*
Chen, P. et al. 2021 [32]	Yes	No	Yes	No	No	No	No	NA	NA	*Acinetobacter*, *Corynebacterium*, *Veillonella*, *Haemophilus*, *Actinomyces*, *Bacterioides*, *Firmicutes*, *Cyanobacteria*, *Fusobacteriota*, *Spirochaetota*, *Phyllobacterium*, *Sphingomonas*
Chen, W. et al. 2021 [33]	Yes	No	No	No	No	No	Yes	NA	NA	*Firmicutes*, *Actinobacteria*, *Fusobacteria*, *Bacteroidetes*, *Acidobacteria*, *Lactobacillus*, *Halomonas*, *Gardnerella*, *Proteobacteria*, *Gemmatimonadetes*, *Patescibacteria*, *Chloroflexi*, *Deinococcus-Thermus*
Lozano, F. et al. 2021 [34]	No	Yes	Yes	No	No	No	Yes	NA	NA	*Anaerobacillus*, *Burkholderia*, *Delftia*, *Dialister*, *Lactobacillus*, *Gardnerella*, *Streptococcus*, *Escherichia*, *Ralstonia*, *Bacillus*
Moreno, I. et al. 2022 [36]	Yes	Yes	Yes	Yes	Yes	Yes	Yes	NA	No	*Anaerococcus*, *Atopobium*, *Bacillus*, *Escherichia*, *Finegoldia*, *Gardnerella*, *Haemophilus*, *Klebsiella*, *Propionibacterium*, *Chryseobacterium*, *Lactobacilllus*, *Micobacterium*, *Streptococcus*, *Haemophilus*, *Staphylococcus*, *Bifidobacterium*, *Cupriavidus*, *Finegoldia*, *Tepidimonas*, *Klebsiella*, *Neisseria*
Chen, Q. et al. 2023 [14]	Yes	Yes	Yes	No	No	Yes	Yes	NA	NA	*Pseudomonas*, *Cutibacterium*, *Methylobacterium*, *Proteobacteria*, *Firmicutes*, *Actinobacteriota*, *Bacteroidota*, *Deinococcota*, *Bdellovibrionota*, *Patescibacteria*, *Cyanobacteria*, *Bdellovibrionota*, *Methylobacterium-Methylorubrum*, *Bradyrhizobium*, *Corynebacterium*, *Prevotella*, *Cutibacterium*, *Deinococcus*, *Brevundimonas*, *Mesorhizobium*, *Acidibacter*, *Peptoniphilus*, *Mesorhizobium*, *Peptoniphilus*
Liang, J. et al. 2023 [38]	Yes	Yes	Yes	No	No	Yes	Yes	NA	NA	*Firmicutes*, *Actinobacteriota*, *Proteobacteria*, *Fusobacteriota*, *Chlamydiae*, *Bacteroidota*, *Lactobacillus*, *Staphylococcus*, *Gardnerella*, *Streptococcus*, *Acinetobacter*, *Ralstonia*
Han, Y. et al. 2024 [40]	No	Yes	Yes	Yes	Yes	No	Yes	NA	No	*Firmicutes*, *Actinobacteriota*, *Proteobacteria*, *Bacteroidota*, *Deinococcota*, *Patescibacteria*, *Fusobacteriota*, *Campilobacterota*, *Cyanobacteria*, *Verrucomicobiota*, *Lactobacillates*, *Bifidobacteriales*, *Enterobacterales*, *Veillonalles-Selenomonadales*, *Coriobacteriales*, *Lachnospirales*, *Peptostreptococcales-Tissieralles*, *Staphylococcales*, *Mycoplasmatales*, *Atopobium*, *Megasphaera*, *Escherichia-Shigella*, *Howardella*, *Blautia*, *Pseudomonas*, *Kocuria*, *Alloscardovia*, *Serratia*, *Faecalibacterium*, *Bifidobacterium*, *Prevotella*, *Gardnerella*
Hmaidan S. et al. 2024 [41]	No	Yes	Yes	Yes	No	No	Yes	NA	Yes	*Streptococcus agalactiae*, *Enterococcus faecalis*, *Gardnerella vaginalis*, *E. coli*, *Staphylococcus aureus*
Kapetanios, V. et al. 2024 [42]	No	Yes	No	Yes	No	No	No	NA	NA	*Enterococcus*, *Staphylococcus* (epidermidis, agalactiae, haemolyticus), *E. coli*, *Proteus*, *Enterobacter cloacae*
Klimaszyk, K. et al. 2025 [45]	No	Yes	Yes	Yes	Yes	Yes	Yes	NA	NA	*Chlamydia*, *Enterobacteriaceae*, *Enterococcus*, *E. coli*, *Gardnerella*, *Klebsiella pneumoniae*, *Mycoplasma*, *Neisseria honorrhoeae*, *Staphylococcus*, *Streptococcus*
Nandagopal, M. et al. 2024 [43]	No	Yes	Yes	Yes	Yes	Yes	No	NA	Yes	*Enterococcus faecalis*, *E. coli*, *Staphylococcus aureus*, *Mycoplasma* spp., *Streptococcus agalactiae*, *Ureaplasma*, *Mycobacterium*, *Chlamydia*
Sanchez-Ruiz, R. et al. 2025 [46]	Yes	Yes	Yes	Yes	No	No	Yes	NA	NA	*Lactobacillus*, *Gardnerella*, *Streptococcus* spp, *Corynebacterium*, *E. coli*, *Bifidobacterium*, *Peptostreptococcus*, *Finegoldia*, *Actinomyces*, *Actinobaculum*, *Gemella*, *Neisseria*, *Prevotella*, *Staphylococcus*, *Parabacterioides*, *Aerococcus*
Takimoto, K. et al. 2023 [39]	Yes	No	Yes	Yes	Yes	No	Yes	NA	NA	*Lactobacillus*, *Ureaplasma*, *Mycoplasma*, *Gardnerella*, *Prevotella*, *Streptococcus*, *Atopobium*, *Dialister*, *Bifidobacterium*, *Anaerococcus*, *Escherischia*, *Enterococcus*
Tanaka, S. et al. 2022 [37]	Yes	No	Yes	Yes	No	No	No	NA	NA	*Lactobacillus*, *Rhodanobacter*, *Atopobium*, *Bifidobacterium*, *Aeromonadaceae*, *Vibrio*, *Clostridiales*, *Burkholderia*, *Streptococcus*
Voropaeva, N. et al. 2021 [35]	Yes	Yes	Yes	Yes	No	No	No	NA	NA	*Lactobacillus* spp., *Enterobacteriaceae*, *E. coli*, *Klebsiella*, *Staphylococcus*, *Streptococcus*, *Candida*
Voroshilina, E. et al. 2020 [31]	Yes	Yes	Yes	Yes	Yes	Yes	Yes	NA	NA	*Lactobacillus*, *Staphylococcus*, *Streptococcus*, *Corynebacterium*, *Gardnerella vaginalis*, *Megasphaera*, *Veillonella*, *Dialister*, *Sneathia*, *Leptotrichia*, *Fusobacterium* spp., *Ureaplasma*, *Mycoplasma hominis*, *Atopobium cluster*, *Bacteroides*, *Porphyromonas*, *Prevotella*, *Anaerococcus*, *Peptostreptococcus*, *Parvimonas*, *Eubacterium*, *Haemophilus*, *Pseudomonas aeruginosa*, *Ralstonia*, *Burkholderia*, *Enterobacteriaceae*, *Trichomonas*, *Neisseria gonorrhoeae*, *Chlamydia*, *Candida*
Zhang, H. et al. 2024 [44]	Yes	No	Yes	No	No	No	Yes	Yes	Doxycycline	*Lactobacillus*, *Proteobacteria*, *Acinetobacter*, *Pseudomonas*, *Gardnerella*, *Phyllobacterium*, *Proteobacteria*, *Bacillus*, *Streptococcus*, *Achromobacter*, *Ralstonia*, *Shewanella*, *Aminicenantales*, *Chloroflexaceae*, *Acinetobacter*

Abbreviations: spp. (species plural).

**Table 4:** Newcastle–Ottawa quality assessment form for cohort studies.

	Selection (Max. 4 Stars)				Comparability (Max. 2 Stars)	Outcome (Max 3 Stars)					
First Author, Year of Publication	Representativeness of Exposed Cohort	Selection of Non-Exposed Cohort	Ascertainment of Exposure	Outcome of Interest not Present at Study Start	Comparability of Cohorts on the Basis of the Design or Analysis Controlled for Confounders	Assessment of Outcome	Sufficient Length of Follow-Up for Outcomes to Occur	Adequacy of Follow-Up of Cohorts	Total	Quality Assessment	Controls
Andrews, W. et al. 2006 [26]	★	★	★	★	★	★		★	7	good	No
Cicinelli, E. et al. 2008 [27]	★	★	★	★		★		★	6	poor	
Cicinelli, E. et al. 2009 [28]	★	★	★	★		★		★	6	poor	No
Cicinelli, E. et al. 2014 [29]	★	★	★	★	★	★		★	7	good	No
Liu, Y. et al. 2019 [30]	★	★	★	★	★	★		★	7	good	
Chen, P. et al. 2021 [32]	★	★	★	★	★	★		★	7	good	
Chen, W. et al. 2021 [33]	★	★	★	★	★	★	★	★	8	good	No
Lozano, F. et al. 2021 [34]	★	★	★	★	★★	★		★	7	good	
Moreno, I. et al. 2022 [36]	★	★	★	★	★	★	★	★	8	good	No
Chen, Q. et al. 2023 [14]	★	★	★	★	★	★		★	7	good	No
Liang, J. et al. 2023 [38]	★	★	★	★	★	★		★	7	good	No
Han, Y. et al. 2024 [40]	★	★	★	★	★	★		★	7	good	
Hmaidan S. et al. 2024 [41]	★	★	★	★	★	★		★	7	good	
Kapetanios, V. et al. 2024 [42]	★	★	★	★	★	★		★	7	good	No
Klimaszyk, K. et al. 2025 [45]	★	★	★	★	★★	★		★	7	good	No
Nandagopal, M. et al. 2024 [43]	★	★	★	★	★	★		★	7	good	
Sanchez-Ruiz, R. et al. 2025 [46]	★	★	★	★	★	★		★	7	good	
Takimoto, K. et al. 2023 [39]	★	★	★	★	★	★		★	7	good	
Tanaka, S. et al. 2022 [37]	★	★	★	★	★	★		★	7	good	No
Voropaeva, N. et al. 2021 [35]	★	★	★	★	★	★		★	7	good	No
Voroshilina, E. et al. 2020 [31]	★	★	★	★	★	★		★	7	good	
Zhang, H. et al. 2024 [44]	★	★	★	★	★	★		★	7	good	No

Good quality: 3 or 4 stars in selection domain AND 1 or 2 stars in comparability domain AND 2 or 3 stars in outcome/exposure domain. Fair quality: 2 stars in selection domain AND 1 or 2 stars in comparability domain AND 2 or 3 stars in outcome/exposure domain. Poor quality: 0 or 1 star in selection domain OR 0 stars in comparability domain OR 0 or 1 stars in outcome/exposure domain. None of the studies proved all samples were negative for CE before analyzing for new cases and controls. Therefore, in the category “selection of non-exposed cohort” all studies were accorded a star. In the category “comparability” only two studies were not accorded any stars because cohort characteristics were not mentioned and two stars were given to studies that matched CE cases with controls. Only two studies explicitly did a follow up. Since for a cross-sectional evaluation of microbiome a single analysis could be considered sufficient follow-up, all studies were starred.

## Data Availability

The current study was based on the results of relevant published studies.

## Supplementary Materials

The following supporting information can be downloaded at https://www.mdpi.com/article/10.3390/biomedicines14040871/s1; Supplementary File S1: Database Searching Strategies: Systematic literature search in Medline, Embase, Web of Science, Scopus, CENTRAL, and Google Scholar; Supplementary File S2: ARRIVE checklist. Reference [60] is cited in the Supplementary Materials.
