# Supplementary material for: The Role of Endometrial Microbiota in the Pathogenesis of Chronic Endometritis: A Systematic Review and Meta-Analysis
Source: Biomedicines. 2026 Apr 10;14(4):871. doi: 10.3390/biomedicines14040871 (PMC13113279; doi:10.3390/biomedicines14040871)
Supplement: Supplementary file 1 [file biomedicines-14-00871-s001.zip › biomedicines-4162308-supplementary (2).pdf]

Systematic literature search

Endometrial Microbiota in Chronic Endometritis

- METHODS –

We conducted a systematic search to identify studies on the uterine microbiome in chronic endometritis. The following bibliographic databases were searched: MEDLINE (Ovid), Embase (Ovid), Web of Science Core Collection, Scopus, CENTRAL (Cochrane Central Register of Controlled Trials), and Google Scholar.

The search strategy combined two key concepts:

- Uterine microbiome (including bacteria)
- Chronic endometritis

For each concept, we used controlled vocabulary terms (MeSH, Emtree) as well as free-text terms (including synonyms and abbreviations). Free-text searches were performed in the fields title, abstract, and keywords.

An initial pilot search in Embase was conducted to test and refine the strategy. In MEDLINE and Embase, animal-only studies were excluded using the Ovid double-negative filter based on indexing terms[1]. In Web of Science and Scopus, animal studies were excluded through keyword filters.

Systematic reviews, meta-analyses, and case reports were excluded either by publication type (MEDLINE) or title terms (Embase). Searches were restricted to publications in English, German, French, and Spanish.

All database searches were conducted on September 24, 2025. The retrieved records were deduplicated using the automated tool Deduklick[2],[3]. An additional four duplicates identified by the screening tool Covidence[4] were removed. The final reference set was imported into Covidence for screening.

---

[1] MEDLINE: https://ospguides.ovid.com/OSPguides/medline.htm, Embase: https://ospguides.ovid.com/OSPguides/embase.htm
[2] https://www.risklick.ch/deduklick
[3] Borissov N, Haas Q, Minder B, et al. Reducing systematic review burden using Deduklick: a novel, automated, reliable, and explainable deduplication algorithm to foster medical research. Syst Rev. 2022;11(1):172. Published 2022 Aug 17. doi:10.1186/s13643-022-02045-9
[4] https://www.covidence.org/

- OVERVIEW –

Search date	Database searched	Platform	Database coverage (inception to present)	Records	Records after deduplication
24 Sep 2025	MEDLINE ALL	Ovid (Wolters Kluwer)	1946 – Sep 23, 2025	757	574
24 Sep 2025	Embase	Ovid (Wolters Kluwer)	1974 – Sep 22, 2025	863	497
24 Sep 2025	Web of Science[5]	Clarivate	1964- present	555	229
24 Sep 2025	Scopus	Elsevier	2004 - present	349	69
24 Sep 2025	Cochrane Central Register of Controlled Trials (CENTRAL)	Wiley	1996 - present	96	42
24 Sep 2025	Google Scholar	Google LLC search via Harzing's Publish or Perish[6]	2004 - present	200	120
**Total references**				2820	**1531**

[5] Web of Science Core Collection containing the following entitlements: Science Citation Index Expanded (SCI-EXPANDED)--1900-present; Science Citation Index Expanded (SCI-EXPANDED)--1900-present; Social Sciences Citation Index (SSCI)--1900-present; Social Sciences Citation Index (SSCI)--1900-present; Arts & Humanities Citation Index (AHCI)--1975-present; Arts & Humanities Citation Index (AHCI)--1975-present; Conference Proceedings Citation Index – Science (CPCI-S)--1990-present; Conference Proceedings Citation Index – Science (CPCI-S)--1990-present; Conference Proceedings Citation Index – Social Science & Humanities; (CPCI-SSH)--1990-present; Conference Proceedings Citation Index – Social Science & Humanities; (CPCI-SSH)--1990-present; Emerging Sources Citation Index (ESCI)--2020-present; Emerging Sources Citation Index (ESCI)--2020-present

[6] Harzing, A.W. (2007) Publish or Perish, available from https://harzing.com/resources/publish-or-perish

- SEARCH STRATEGIES -

MEDLINE (Ovid)
Search date: September 24, 2025

Ovid MEDLINE(R) ALL <1946 to September 23, 2025>

1 ((microbi* or micro-bi* or flora or microflor* or ecosystem* or microorgan* or micro-organ* or polymicrobi* or poly-microbi* or bacteri* or bacteroid* or proteobacter* or proteo-batcter* or eubacteria* or clostridi* or staphylococc* or actinobacter* or actino-bacter* or firmicute* or lactobacil* or nonlactobacil* or non-lactobacil* or ralstoni* or gardnerel* or ureaplasm* or chlamydi* or Escherichi* or enterococc* or fusobacteri* or prevotell* or atobiu* or cultur*) adj5 (uter* or endometri* or intrauter* or intra-uter*)).ti,ab,kf. 6321

2 (exp Microbiota/ or exp Bacteria/) and exp Uterus/ 3700

3 1 or 2 9255

4 (((chronic or inflamm* or persist*) adj6 (endometrit* or endometrial* or (uter* adj4 cavit*) or (inner layer* adj4 uter*) or endometrium or uterin* or intrauter* or intra-uter*)) or endomyometrit*).ti,ab,kf. 7022

5 exp Endometritis/ 4764

6 4 or 5 10641

7 3 and 6 1290

8 (exp animals/ or exp animal experimentation/ or exp models, animal/ or exp plants/ or exp fungi/) not humans/ 5842523

9 7 not 8 684

10 (case reports or "systematic review" or meta analysis).pt. 2892292

11 9 not 10 622

12 limit 11 to (english or french or german or spanish) 575

Link to search strategy

****

Embase (Ovid)
Search date: September 24, 2025

Embase <1974 to 2025 September 22>

1 ((microbi* or micro-bi* or flora or microflor* or ecosystem* or microorgan* or micro-organ* or polymicrobi* or poly-microbi* or bacteri* or bacteroid* or proteobacter* or proteo-batcter* or eubacteria* or clostridi* or staphylococc* or actinobacter* or actino-bacter* or firmicute* or lactobacil* or nonlactobacil* or non-lactobacil* or ralstoni* or gardnerel* or ureaplasm* or chlamydi* or Escherichi* or enterococc* or fusobacteri* or prevotell* or atobiu* or cultur*) adj5 (uter* or endometri* or intrauter* or intra-uter*)).ti,ab,kf. 7912

2 (exp bacterium/ or exp microbiome/) and exp uterus/ 4634

3 1 or 2 11484

4 (((chronic or inflamm* or persist*) adj6 (endometrit* or endometrial* or (uter* adj4 cavit*) or (inner layer* adj4 uter*) or endometrium or uterin* or intrauter* or intra-uter*)) or endomyometrit*).ti,ab,kf. 10339

5 exp Endometritis/ 9193

6 4 or 5 17401

7 3 and 6 1819

8 (exp animal/ or exp invertebrate/ or nonhuman/ or animal experiment/ or animal tissue/ or animal model/ or exp plant/ or exp fungus/) not (exp human/ or human tissue/) 8404057

9 7 not 8 958

10 ((systematic adj3 review*) or meta-analys* or metaanalys* or case report*).ti. 966718

11 9 not 10 933

12 limit 11 to (english or french or german or spanish) 863

Link to search strategy

****

Web of Science
Search date: September 24, 2025

1 TS=((microbi* OR micro-bi* OR flora OR microflor* OR ecosystem* OR microorgan* OR micro-organ* OR polymicrobi* OR poly-microbi* OR bacteri* OR bacteroid* OR proteobacter* OR proteo-batcter* OR eubacteria* OR clostridi* OR staphylococc* OR actinobacter* OR actino-bacter* OR firmicute* OR

lactobacil* OR nonlactobacil* OR non-lactobacil* OR ralstoni* OR gardnerel* OR ureaplasm* OR chlamydi* OR Escherichi* OR enterococc* OR fusobacteri* OR prevotell* OR atobiu* OR cultur*) NEAR/5 (uter* OR endometri* OR intrauter* OR intra-uter*))
Results: 7374

2 TS=((((chronic OR inflamm* OR persist*) NEAR/6 (endometrit* OR endometrial* OR (uter* NEAR/4 cavit*) OR ("inner layer*" NEAR/4 uter*) OR endometrium OR uterin* OR intrauter* OR intra-uter*)) OR endomyometrit*))
Results: 7884

3 #1 AND #2
Results: 862

4 #1 AND #2 and Veterinary Sciences or Agriculture Dairy Animal Science or Zoology or Agronomy or Fisheries or Agriculture Multidisciplinary or Agricultural Engineering (Exclude – Web of Science Categories) and English or German or French or Spanish (Languages)
Results: 555

Link to search strategy

****

Scopus
Search date: September 24, 2025

(TITLE-ABS-KEY ((microbi* OR micro-bi* OR flora OR microflor* OR ecosystem* OR microorgan* OR micro-organ* OR polymicrobi* OR poly-microbi* OR bacteri* OR bacteroid* OR proteobacter* OR proteo-batcter* OR eubacteria* OR clostridi* OR staphylococc* OR actinobacter* OR actino-bacter* OR firmicute* OR lactobacil* OR nonlactobacil* OR non-lactobacil* OR ralstoni* OR gardnerel* OR ureaplasm* OR chlamydi* OR Escherichi* OR enterococc* OR fusobacteri* OR prevotell* OR atobiu* OR cultur*) W/5 (uter* OR endometri* OR intrauter* OR intra-uter*))) AND (TITLE-ABS-KEY (((chronic OR inflamm* OR persist*) W/6 (endometrit* OR endometrial* OR (uter* W/4 cavit*) OR ("inner layer*" W/4 uter*) OR endometrium OR uterin* OR intrauter* OR intra-uter*)) OR endomyometrit*)) AND (EXCLUDE (EXACTKEYWORD , "Nonhuman") OR EXCLUDE (EXACTKEYWORD , "Animals") OR EXCLUDE (EXACTKEYWORD , "Animal") OR EXCLUDE (EXACTKEYWORD , "Cattle") OR EXCLUDE (EXACTKEYWORD , "Animal Model") OR EXCLUDE (EXACTKEYWORD , "Bovine") OR EXCLUDE (EXACTKEYWORD , "Animal Cell") OR EXCLUDE (EXACTKEYWORD , "Mouse") OR EXCLUDE (EXACTKEYWORD , "Cattle Diseases") OR EXCLUDE (EXACTKEYWORD ,

"Cattle Disease") OR EXCLUDE (EXACTKEYWORD , "Veterinary Medicine") OR EXCLUDE (EXACTKEYWORD , "Horse") OR EXCLUDE (EXACTKEYWORD , "Horses") OR EXCLUDE (EXACTKEYWORD , "Animal Disease") OR EXCLUDE (EXACTKEYWORD , "Mice") OR EXCLUDE (EXACTKEYWORD , "Horse Diseases") OR EXCLUDE (EXACTKEYWORD , "Mare") OR EXCLUDE (EXACTKEYWORD , "Horse Disease") OR EXCLUDE (EXACTKEYWORD , "Dairy Cattle") OR EXCLUDE (EXACTKEYWORD , "Rat")) AND (LIMIT-TO (LANGUAGE , "English") OR LIMIT-TO (LANGUAGE , "German") OR LIMIT-TO (LANGUAGE , "French") OR LIMIT-TO (LANGUAGE , "Spanish"))

Results 349

****

Cochrane Library (CENTRAL)
Search date: September 24, 2025

#1 ((microbi* OR micro-bi* OR flora OR microflor* OR ecosystem* OR microorgan* OR micro-organ* OR polymicrobi* OR poly-microbi* OR bacteri* OR bacteroid* OR proteobacter* OR proteo-batcter* OR eubacteria* OR clostridi* OR staphylococc* OR actinobacter* OR actino-bacter* OR firmicute* OR lactobacil* OR nonlactobacil* OR non-lactobacil* OR ralstoni* OR gardnerel* OR ureaplasm* OR chlamydi* OR Escherichi* OR enterococc* OR fusobacteri* OR prevotell* OR atobiu* OR cultur*) NEAR/5 (uter* OR endometri* OR intrauter* OR intra-uter*)):ti,ab,kw 481

#2 ([mh Microbiota] OR [mh Bacteria]) AND [mh Uterus] 69

#3 #1 OR #2 493

#4 (((chronic OR inflamm* OR persist*) NEAR/6 (endometrit* OR endometrial* OR (uter* NEAR/4 cavit*) OR (("inner" NEXT layer*) NEAR/4 uter*) OR endometrium OR uterin* OR intrauter* OR intra-uter*)) OR endomyometrit*):ti,ab,kw 608

#5 [mh Endometritis] 338

#6 #4 OR #5 877

#7 #3 AND #6 in Trials 96

Link to search strategy

****

Google Scholar (via Harzing's Publish or Perish)
Search date: September 24, 2025

Keyword search, include citations, limited to the 200 most relevant records:

(("chronic endometritis"|"chronic intrauterine inflammation") AND ("uterine microbiome"|"endometrial microbiome"|"intrauterine microbiome|"uterine bacteria"|"endometrial bacteria"|"intrauterine bacteria")) NOT (cattle|horse|veterinary)

Results 200
